# Disruption of cardio-pulmonary coupling in myopathies: Pathophysiological and mechanistic characterization with special emphasis on nemaline myopathy

**DOI:** 10.3389/fcvm.2022.996567

**Published:** 2022-11-07

**Authors:** Diana Maria Ronderos-Botero, Arundhati Dileep, Laura Yapor, Ravish Singhal

**Affiliations:** Division of Pulmonary and Critical Care Medicine, Department of Medicine, BronxCare Health System, The Bronx, NY, United States

**Keywords:** heart failure, respiratory dysfunction, heart-lung interaction, myopathy, nemaline myopathy

## Abstract

The heart and lung are in continuous reciprocal interaction that creates a functional and anatomical reserve referred to as cardiopulmonary coupling (CPC). Disruption of CPC can occur due to various cardiac or pulmonary pathologies but also can occur in patients with myopathies. Nemaline myopathy (NM) is a skeletal muscle heterogeneous disorder due to contractile proteins' gene mutations that impact lung and cardiac mechanics and thus is expected to adversely affect CPC in a complex manner. We present a case of NM and we review the literature on cardiac and pulmonary effects of myopathy-related disruption of CPC.

## Case illustration

A 43-year-old man presented to the emergency department with generalized fatigue and respiratory distress. The patient reported 3–4 weeks of gradually worsening fatigue associated with dry cough and night sweats. He denied chest pain, fever, or leg edema. His past medical history includes non-insulin-dependent diabetes mellitus and hypertension. He was diagnosed with nemaline myopathy at the age of 10 *via* muscle biopsy that showed rounded and atrophic type 1 muscular fibers. Gomori stain showed numerous predominantly type 1 and some type 2 muscle fibers with nemaline rods. His phenotypic characteristics at that time were normal ocular muscles and ogival palate; however, there was discrete facial palsy and paresis of the vocal cords with bilateral weakness of sternocleidomastoid and respiratory muscles.

His electrocardiogram (ECG) at 11 years old revealed right axis deviation, right bundle branch block, and moderate right ventricular hypertrophy. Prior chest X-Ray showed a Cardiothoracic index of 0.50 and normal aorto-pulmonary arch. Despite having upstream and downstream muscle weakness, he was able to perform basic daily life activities. He followed closely with neurology and physical therapy and focused on breathing exercises. His disease was characterized as slowly progressive but with an unpredictable course.

**Figure 1:**
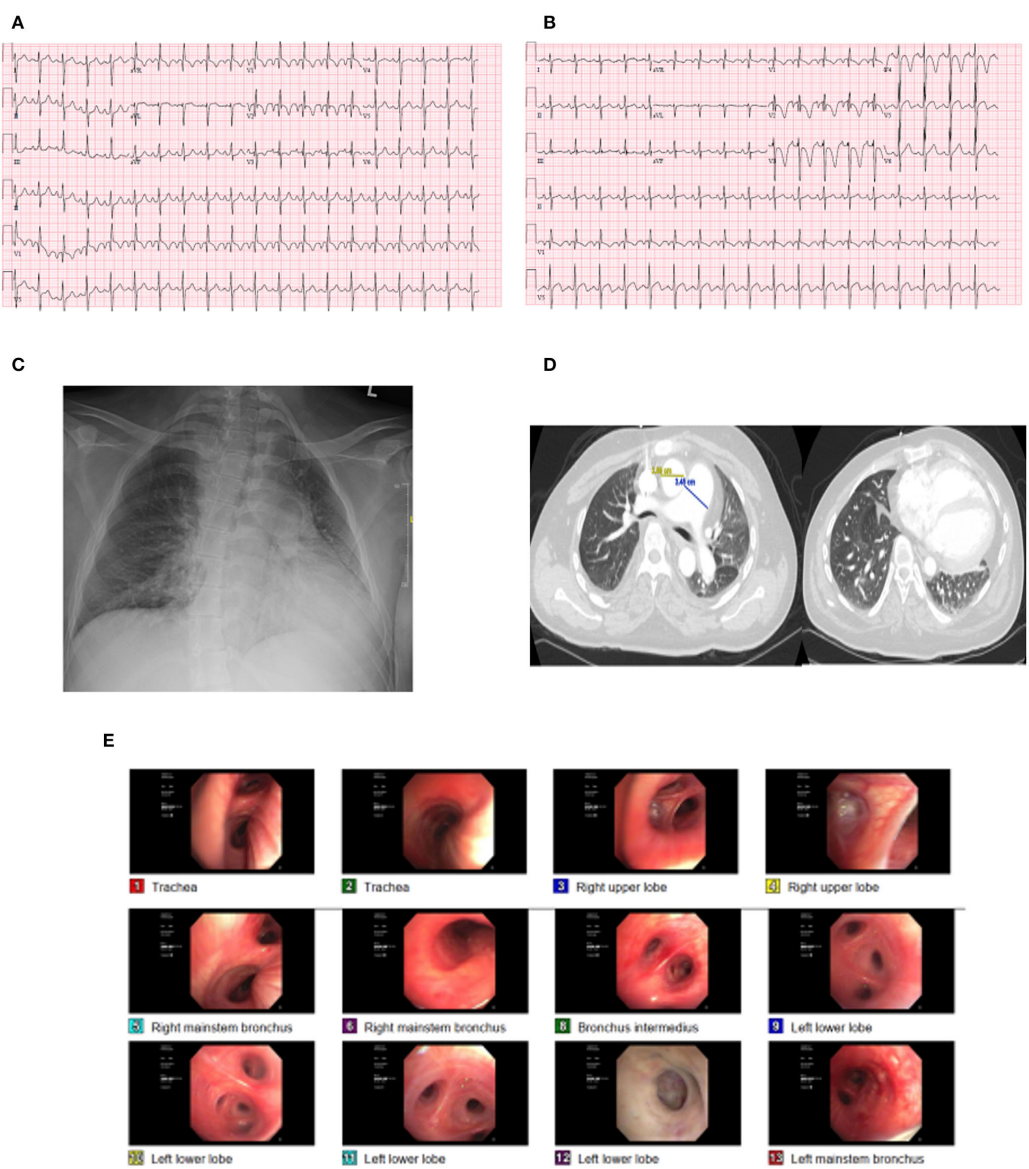
Case illustration. **(A)** initial ECG showing sinus tachycardia and right bundle branch block. **(B)** repeat ECG showing ST depressions in V1 to V4. **(C,D)** CXR and CT of the chest showing pulmonary congestion and ground glass opacity. **(E)** Bronchospcopy.

On admission, he was in moderate respiratory distress with tachycardia (122 beats per minute) and tachypnea (25 cycles per minute) with oxygen saturation of 88% on room air. A cardiac examination revealed no obvious heart murmurs and the lungs were clear to auscultation. His admission ECG (Figures 1A,B) showed sinus tachycardia, left atrial enlargement, right axis deviation, incomplete right bundle branch block, and right ventricle hypertrophy. Chest X-ray (Figure 1C) showed perihilar interstitial prominence, small to moderate left pleural effusion, mild cardiomegaly, and dextroscoliosis of the thoracic spine.

The patient was obtunded and was started on oxygen by nasal cannula and his arterial blood gas (ABG) showed a pH of 7.31, PaCO_2_ 143, and PaO_2_ 110. Computed tomography of the chest (Figure 1D) with contrast showed no pulmonary embolism and diffuse ground glass opacities suggestive of pulmonary vascular congestion. His laboratory workup revealed hemoglobin 16.2 g/dl, bicarbonate of 40 mEq/L, brain natriuretic peptide of 1,976 pg/mL, and slight troponin elevation (high sensitivity troponin: first value 14 ng/L, repeat value 17 ng/L).

The patient was admitted to the medical intensive care unit (ICU) and was placed on mechanical ventilation and was started on antibiotics. He was found to have low static lung compliance of 28.6 cm H_2_O (normal value: 60–100 cm H_2_O). Repeat CXR revealed persistent pulmonary infiltrates, and accordingly, the patient underwent bronchoscopy (Figure 1E), which showed no endobronchial lesions, normal mucosa, or scant mucoid secretions. Bronchoalveolar lavage showed WBC 245 cells/mm^3^ and 95% of segmented cells. All cultures and gram were negative. He underwent an echocardiogram, which showed an ejection fraction of 46%, concentric left ventricular hypertrophy with global left ventricular hypokinesis, normal right ventricular (RV) function, normal RV tissue Doppler systolic velocity 12 cm/s, RV tricuspid annular plane systolic excursion (TAPSE) of 2.14 cm, and enlarged right atrium. There was no evidence of pericardial effusion, and RV systolic pressure was not possible due to inadequate tricuspid regurgitation jet. Intravenous diuresis was started for management of heart failure exacerbation with good urine output, and on the second day, the patient's respiratory and hemodynamic status improved, and was liberated off the ventilator to noninvasive ventilation and eventually to a nasal cannula and was discharged to the floors where he completed the course of antibiotics. He was discharged and was followed in the pulmonary clinic where he underwent a pulmonary function test which revealed concomitant obstruction and extrathoracic lung restriction. The maximum voluntary ventilation (MVV) value was 65% (135% of predicted) demonstrating no muscle weakness at that time. The patient was planned for a repeat echocardiogram, cardiac catheterization to assess coronary anatomy, and a sleep test to rule out sleep apnea as part of his chronic respiratory failure and the high prevalence of sleep apnea and muscle disease; however, the patient was reluctant to pursue further testing and was lost to follow up.

## Introduction

Heart and lungs are highly anatomically and functionally related and are tightly interdependent. The realization of the co-existence of cardiopulmonary interactions and the understanding of this vital codependency continued to evolve for the past 2,000 years. The credit for this discovery is distributed in history from the time of Hippocrates in the 4th century BC to William Harvey and Marcelo Malpighi in the 17th century AD when the full description was finalized. The first intelligible description of the cardiopulmonary interaction was established by Ibn Nafis (Annafis, 1,210–1,288 AD) in his most famous book Sharah al Tashreeh al Qanoon (Commentary on the anatomy of the Canon of Avicenna) where he described for the first time the pulmonary circulation as a connection between the right and left heart rather than a direct intracardiac connection (1).

The heart and lung are in continuous reciprocal interaction that creates a functional and anatomical reserve referred to as cardiopulmonary coupling (CPC), which is a physiological determinant of cardiopulmonary function as well as a pathological contributor to disease processes. Reportedly, CPC can be disrupted at either the respiratory side or the cardiac side. Primary myopathies are associated with pulmonary and cardiac abnormalities that can affect cardiopulmonary function leading to significant morbidities and mortality. As such, myopathies can also disrupt CPC (2); however, the underlying mechanism of CPC in the progression of myopathies is not well described.

Classically, hypoventilation seems to be a primary pathophysiological mechanism that disrupts CPC in patients with myopathy. Hypoventilation can occur in a plethora of diseases; however, it is known to commonly occur in diseases affecting neuromuscular junction and respiratory muscles such as primary myopathies due to attenuation of diaphragmatic and inspiratory muscles and further resulting in a mismatch between ventilation and perfusion (V/Q) (3). Myopathy-related V/Q mismatch is characterized by high PaCO_2_ levels, and in the presence of concomitant cardiac or pulmonary diseases, hypoxemia can also occur and can be severe.

Nemaline myopathy (NM) is a skeletal muscle heterogeneous disorder that is histologically characterized by the presence of nemaline inclusion bodies within the muscle fibers.

NM is a rare disease the prevalence of which in all age groups ranges from 0.14 to 0.26 per 100,000 and is diagnosed with the combination of clinical presentation and muscle biopsy. Patients with the congenital typical form of NM, initially present with proximal (upstream) and later distal (downstream) progressive muscle weakness (4). NM is clinically heterogeneous, and at least six types of clinical manifestations have been described including respiratory muscle weakness causing hypoventilation and hypercapnia (5). Cardiac involvement is rare, however, is reported (6), including ischemic and valvular heart disease, and cor-pulmonale, and could potentially be exacerbated by the progression of the myopathy. NM affects the lung's mechanics and is thus expected to adversely affect CPC in a complex manner (7). A combination of acute or chronic heart failure associated with acute or chronic respiratory failure can occur and could be attributed to either cardiac or respiratory affections. A mixed cardiopulmonary involvement is rare in NM, especially when other etiologies for respiratory and heart failure had been ruled out as illustrated in our case.

In this review, we will briefly discuss the concept of cardiopulmonary coupling and its potential effect on the pathophysiology of myopathies with special emphasis on Nemaline Myopathy (NM) with a mixed respiratory and cardiac failure. We will also describe a distinct clinical phenotype specific to patients with skeletal muscle myopathies after other etiologies for heart or respiratory failure have been ruled out. Putting that together, our proposed phenotype in addition to the two previously described phenotypes in patients with myopathy will be emphasized and serve as a road map toward recognition and early detection that could critically affect the management of patients with myopathies.

## Concept of cardiopulmonary coupling

The current evolved models of cardiopulmonary interactions have a level of complexity that is beyond the simple understanding of ventilation/perfusion/pump models (8). Although cardiopulmonary interactions in healthy individuals are generally considered to minimally affect hemodynamics during rest, it functions at its steep part in physiological conditions, such as exercise and sleep, and other pathological processes such as myocardial dysfunction, pulmonary hypertension, lung parenchymal diseases, airway diseases, and myopathies, among others (Supplementary Table 1).

The reciprocal nature of the co-expression of arterial pulse and respiratory rhythms indicates, at least, a middle ground of coupled control (9). Cardiorespiratory coupling (CRC) results from shared complementary functions affecting heart rate (HR), blood pressure (BP), and ventilation rhythms (10, 11). These factors can be categorized into neuronal factors, reflex factors, central factors, and chemoreceptors (Supplementary Table 1, Supplementary Figure 1) (8). Moreover, CPC is also a function of mechanical coupling at the level of ventricular interdependence that is affected by changes in the thoracic pressure during respiration, and connections of the right heart to the precapillary component of the pulmonary circulation and the left heart to the postcapillary component of the pulmonary circulation. These factors add complex mechanisms by which CPC can mediate ventilation, perfusion, cardiac diastolic, and systolic performance (Supplementary Table 1, Supplementary Figure 2).

It should be noted that changes in intrathoracic pressures are tightly related to healthy respiratory muscular contractions.

## Ventilation/perfusion: The functional crossroads of the cardiopulmonary coupling

The common functional point of cardiopulmonary interaction is the gas exchange that fulfills blood flow or what is known as a ventilation/perfusion match (Supplementary Figure 3) (12).

Gas exchange depends greatly on the matching of perfusion and ventilation (V/Q). Essentially, V/Q is the ratio of the amount of air that reaches the lungs (alveolar ventilation) divided by the amount of blood flow in the pulmonary capillaries (capillary perfusion).

V/Q mismatch is caused by either pathological decreased perfusion (dead space physiology) or decreased ventilation (shunt physiology). Pathological dead space occurs when the amount of blood flow does not match the required ventilation in conditions, such as cardiogenic shock, emphysema, and pulmonary embolism, and pathological shunting occurs due to the direct passage of blood from the veins to the right heart bypassing a segment of the lung or all pulmonary circulation, such as congenital cardiac shunts and atrioventricular malformations, or pathological hypoventilation that occurs in conditions such as pneumonia, pulmonary edema, tissue trauma, atelectasis, mucous plugging, post-operative pain and effect of some drugs, and anesthetics that decrease peripheral or central respiratory drive.

Importantly, intrapulmonary arteries constrict in response to alveolar hypoxia, or what is called hypoxic pulmonary vasoconstriction in a trial to minimize shunting by diverting blood to better-oxygenated lung segments, thereby optimizing ventilation/perfusion matching and systemic oxygen delivery (13).

Consequently, V/Q mismatch can occur due to pulmonary pathologies and cardiac pathologies. Pulmonary pathologies, such as chronic lung diseases, can involve all components of the respiratory system, including the airways, the pulmonary vasculature, and the alveolar-capillary interface, resulting in resting hypoxemia (14).

From the cardiac side, right or left ventricular dysfunction can impair cardiac-output capability, leading to impaired oxygen delivery and early development of lactic acidosis. The presence of pulmonary vascular abnormalities, pulmonary hypertension, and right-ventricular dysfunction can affect V/Q ratios causing hypoxemia (14).

Muscle relaxation or muscle disease, which attenuates or entirely removes diaphragmatic and inspiratory muscle tone, further alters the distribution of ventilation. This results in V/Q mismatch due to hypoventilation (3). Hypoventilation can occur in a plethora of diseases however is known to commonly occur in diseases affecting neuromuscular junction such as Myasthenia gravis, Lambert–Eaton syndrome, and diseases affecting respiratory muscles such as primary myopathies and chest wall deformities.

The hallmark of myopathy-related V/Q mismatch is hypoventilation causing a high PaCO_2_ level. Hypoventilation in myopathic patients does not produce significant hypoxemia in the healthy lung, but in the presence of concomitant cardiac or pulmonary diseases, hypoxemia can be severe. One characteristic feature of hypoventilation-induced hypoxemia is that it is easily correctible by supplemental oxygen, a feature that occurred in the patient illustrated above however without correction of hypercapnia that was the primary effect of hypoventilation due to myopathic effect of the respiratory muscles.

## Myopathies related to cardiopulmonary pathologies

Although primary myopathies encompass a grand diversity of entities, most of them share cardiac and respiratory involvement and impairment. Skeletal muscles require a large ATP pool to meet basic energy requirements at rest (15), and this ATP production rate can increase to approximately 100-folds during exercise (16). The different forms of inflammatory and primary myopathies can manifest differently. Symptoms can be expressed as myalgias, easy fatigability, fasciculations, scapular winging, dysphagia, dysarthria, eyelid drop, gait disturbances, falls, muscle rigidity, muscle cramps, dyspnea, myoglobinuria, and rhabdomyolysis among others.

Cardiac involvement includes cardiomyopathies, valvular insufficiencies, intracardiac thrombosis, or diastolic dysfunction (Table 1).

**Table 1:** Cardiac and pulmonary effects in different primary myopathies.

**Classification**	**Example**	**Cardiac effects**	**Pulmonary effects**
Dystrophies	Duchenne muscular dystrophy	•Supraventricular and ventricular arrhythmias •Abnormal Q waves, ST segment depression, •Prolonged QT interval, •Myocardial thickening, •Regional wall motion abnormalities, •Dilatation, secondary Valve insufficiency, •Heart failure with systolic and diastolic dysfunction •Reduction of the coronary vasodilative reserve, hypercoagulability	•respiratory weakness
	Emery-dreifuss muscular dystrophy (EDMD)	•Prolonged PQ interval •Conduction defects •Sudden death •Dilatated Cardiomyopathy	•Occurs in association with skeletal deformities
	Facioscapulohumeral muscular dystrophy (FSH)	•Intraventricular conduction delay •Supraventricular arrhythmias •Ventricular tachycardia, Atrioventricular block •Long QT syndrome, •Abnormal nuclear myocardial perfusion •Myocardial thickening	•Respiratory insufficiency reported in severe disease
	Limb girdle muscular dystrophies (LGMD)	•Incomplete right bundle branch block •Shortened QT interval •Reduction of the coronary vasodilative reserve •Dilated cardiomyopathy	•Can occur due to scoliosis and spinal rigidity
	Congenital muscular dystrophies	•Reports of myocardial fibrosis •Reports of heart failure	•Infantile/childhood: Inevitable •Adult: Common
	Type 1 myotonic dystrophy	•AV conduction abnormalities •QT interval •Torsades de pointes •Myocardial thickening •Heart failure	•Sleep disordered breathing •Respiratory muscle weakness and myotonia •Alveolar hypoventilation •Aspiration pneumonia
	Type 2 myotonic dystrophy	•Supraventricular tachycardia, •Ventricular ectopy •Atrial fibrillation •Myocardial infarction	
	Proximal myotonic myopathy (PROMM)	•Sinus bradycardia •Abnormal T waves •Right bundle branch block •Sustained monomorphic ventricular tachycardia	
Metabolic myopathies	Glycogen storage diseases	•Myocardial thickening •Heart failure •Short PR	•Respiratory failure with acidosis reported
	Fatty acid oxidation defects	•Myocardial thickening	•Uncommon unless severe rhabdomyolysis
	Mitochondriopathie	•Myocardial thickening •Cardiomyopathy	•Respiratory muscle weakness •Hyperventilation syndrome secondary to acidosis Central hypoventilation
Disorders of the contractile proteins	Desmin myopathy	•Atrial flutter •AV block •Ventricular tachycardia	
		•Myocardial thickening •Cardiomyopathy •Heart failure with systolic and restrictive diastolic dysfunction •Right ventricular dysfunction	
	Nemaline myopathy	•Myocardial thickening •Dilated cardiomyopathy •Heart failure	•Infantile/childhood: Inevitable •Adult: Common
	Central Core disease	•Myocardial thickening •Dilated cardiomyopathy •Heart failure	•Rare
Nonprogressive congenital myopathies		•Atrial fibrillation •AV conduction abnormality •Dilated cardiomyopathy •Heart failure	
Unclassified myopathies	Barth syndrome	•Endocardial fibroelastosis •Increased number of mitochondria in cardiomyocytes •Myocardial thickening	
	Bethlem myopathy	•Myocardial thickening	•Rare
	McLeod syndrome	•progressive heart disease	

Several primary myopathies exhibit significant cardiac and respiratory involvement. Examples of such primary myopathies include (Table 1):

a) *Dystrophies* (17): genetic diseases that cause progressive muscle weakness and degeneration during voluntary movement that vary in age of onset, severity, and pattern of affected muscles. Examples include Duchenne muscular dystrophy, Emery-Dreifuss Muscular Dystrophy (EDMD), Facioscapulohumeral Muscular Dystrophy (FSH), Limb Girdle Muscular Dystrophies (LGMD), Congenital Muscular Dystrophies, Type 1 and Type 2 Myotonic Dystrophy (MD2), and Proximal (upstream) Myotonic Myopathy (PROMM).
b) *Metabolic Myopathies* (18): a group of myopathies that vary clinically and etiologically that are caused by defects in cellular energy metabolism. Such defects can be classified into glycogen storage diseases (Glycogenosis), fatty acid oxidation defects such as Very-Long-Chain Acetyl-CoA Dehydrogenase Deficiency, Myoadenylate Deaminase Deficiency (MADAD), Systemic Carnitine Deficiency (SCD), and Lysosomal Glycogen Storage with Normal Acid Maltase Activity, and mitochondrial disorders due to respiratory chain impairment (Mitochondriopathies).
c) *Disorders of the Contractile Proteins* (19): a group of diseases characterized by mutations in several muscle structural proteins (the myosin heavy chain, tropomyosin, cardiac troponin T, and myosin binding protein C). Of note, these mutations are widely known to be associated with cardiac abnormalities. Examples include Desmin Myopathy which is characterized by abnormal aggregates of desmin-type intermediate filaments in the skeletal, cardiac, and rarely the intestinal smooth muscle, Nemaline Myopathy (NM) is a rare, genetically heterogeneous, autosomal dominant, or recessive myopathy due to mutations in the alpha-actin, alpha-tropomyosin or nebulin gene, and Central Core Disease (CCD).

## Myopathies' effect on the cardiovascular system

**Figure 2:**
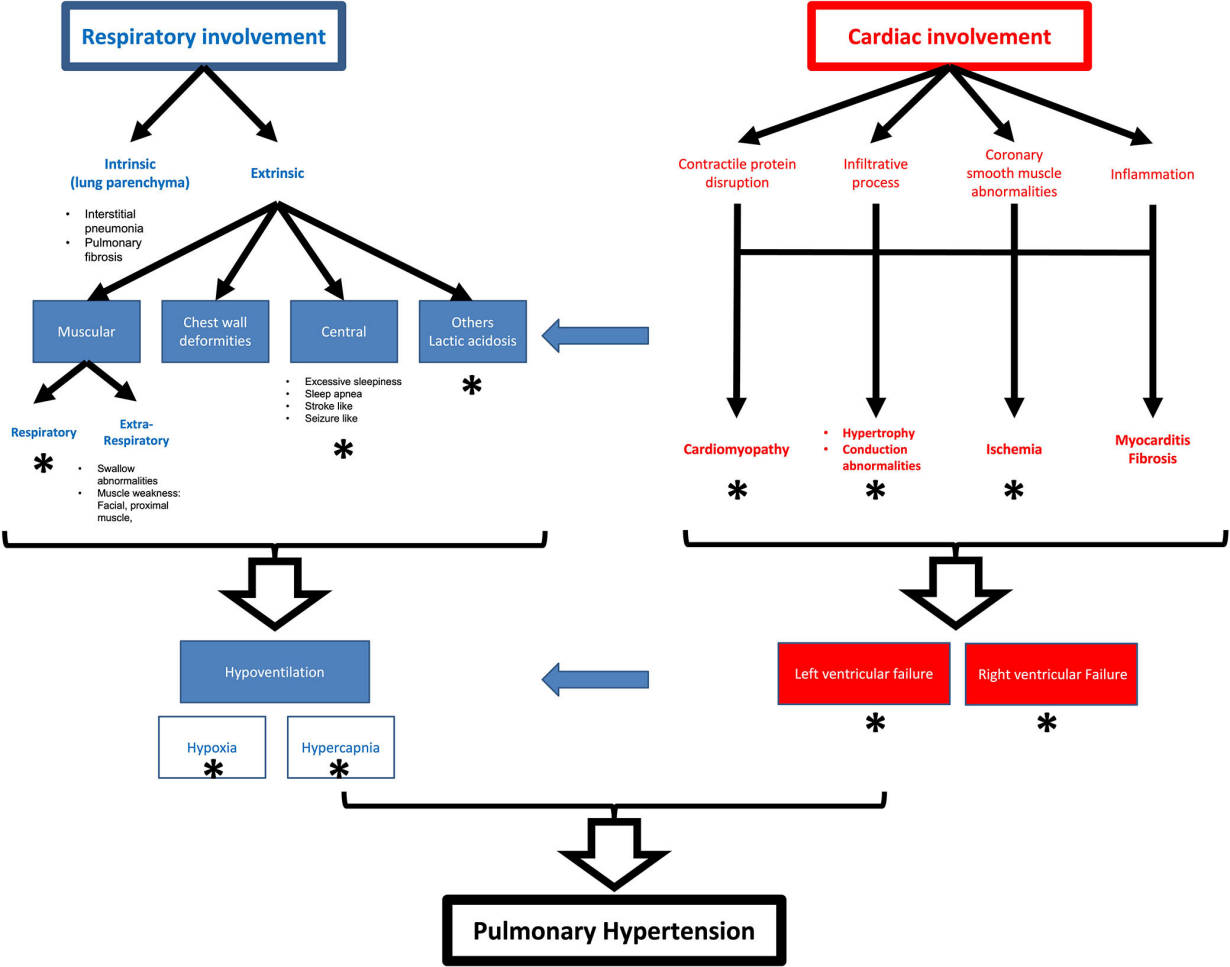
Schematic representation of pulmonary and cardiac effects in myopathies and their relationships. Star represent mechanisms reported for nemaline myopathy.

There is a wide variety of cardiac involvement in primary myopathies that affect all parts of the heart isolated or combined including the myocardium, the conduction system, and the valvular apparatus (Table 1, Figure 2). The mechanisms and pathogenesis of cardiac involvement in myopathic patients are controversial and do not follow a uniform scheme. Many mechanisms have been described including gene mutation disrupting the cardiac contractile proteins, coronary smooth muscle abnormalities causing abnormal coronary reserve and myocardial ischemia, abnormal protein depositions causing conduction defects, restrictive and hypertrophic cardiomyopathies, myocardial inflammation, and fibrosis causing cardiomyopathies and arrythmia (Table 2, Figure 2).

**Table 2:** Cardiac and pulmonary pathophysiological mechanisms in myopathies.

**Cardiac effects**	**Mechanism**	**Manifestation**	**Effect**
Blood flow	Impaired myocardial perfusion Impaired coronary flow reserve	ST-T changes	Ischemia, inflammation, increased LV pressures, arrhythmia
	Inflammation	Inflammatory cells infiltrates Increased inflammatory markers	Myocarditis
Functions	Heart Failure	Decreased ejection fraction Dilated Chambers	Increased Myocardial wall stress Increased postcapillary pulmonary pressures Reactive pulmonary vasoconstriction → increased PVR Hypoxia Pulmonary edema
Myocardial Structures	Myocardial fibrosis	Myocardial stiffness Electrical anisotropy Conduction abnormalities	Increased LV pressures, arrhythmia, AV blocks
Pulmonary effects			
Intrinsic	Lung parenchymal involvement causing either bronchiolitis, diffuse alveolar hemorrhage, NSIPor UIP or pulmonary fibrosis	Hypercapnia and hypoxia	Pulmonary vasoconstriction → increased PVR muscularization of the venules → increased PVR secondary erythrocytosis Acidosis → increased PVR
Extrinsic	Weakness in primary respiratory muscles	Hypercapnia and hypoxia	Derecruitment of the vasculature → worsening hypoxia Increase Intrapleural pressures → decrease venous return
	Thoracic cavity deformities	Hypercapnia and hypoxia	Extrathoracic lung restriction Respiratory acidosis
Mixed	V/Q mismatch	Hypoventilation	High PaCO2 level as adequate In the presence of lung diseases, hypoxemia (correctible by supplemental oxygen).

The concomitant respiratory muscles involvement with accompanying hypoventilation and pre-capillary pulmonary hypertension may occur causing pulmonary hypertension and strain on the right side of the heart causing right ventricular involvement that may be isolated in some cases.

Despite that there are no specific myopathic ECG patterns, however, ECG manifestations can signify abnormalities in impulse generation and conduction defects. All types of heart block, bradycardia, and supraventricular and ventricular arrhythmias are described. Myocardial involvements include focal myocardial thickening, cardiac chambers dilation, Takotsubo cardiomyopathy, left-ventricular hyper-trabeculation, apical hypertrophic cardiomyopathy, and dilated or restrictive cardiomyopathy. Similarly, there is no specific cardiac targeted treatment in patients with myopathy. Therapies that can be used include guideline-directed medical therapies for heart failure, pacemakers, intracardiac defibrillators, and high radiofrequency ablation for tachycardia, all the way up to advanced therapies, such as ventricular assist devices and cardiac transplantation.

The specific prognosis in patients with cardiac involvement complicating myopathy is usually fair or similar to the general prognosis of cardiac patients. The prognosis in general is favorable if cardiac involvement is established early and adequately managed. However, some manifestations of cardiac involvement in myopathy carry worse prognoses by nature such as ventricular arrhythmias, intractable heart failure not amenable to adequate drug treatment, in the absence of a heart donor, or contraindications for a CRT system or transplantation.

### Cardiovascular manifestations of specific myopathies

#### Muscular dystrophies

The frequency and type of cardiac involvement vary in muscular dystrophies. The muscular dystrophies with the most common cardiac involvement are DMD, EDMD, and LGMD.

In dystrophinopathies (DMD), cardiac involvement is an important component of DMD and generally occurs in up to 90% of the patients and up to 50% of the carriers. Cardiac involvement includes supraventricular and ventricular arrhythmias, abnormal Q waves, ST segment depression, prolonged QT interval, myocardial thickening, regional wall motion abnormalities, dilatation, secondary valve insufficiency, and heart failure with systolic and diastolic dysfunction (20, 21). In several DMD cases, reduction of the coronary vasodilative reserve, possibly due to the involvement of the vascular smooth muscle, has been described (22). The degree of cardiac involvement in female carriers is dependent on the amount of X-chromosome inactivation. And range from asymptomatic to severely affected.

The Cardiac abnormalities in EDMD are dominant features of the disease that occur by adulthood. In EDMD patients exhibit bradycardia, conduction blocks that may life-threatening causing sudden death making it necessary to insert permanent (23). Dilated myopathy can occur however is rare.

In LGMD, 20% of patients may exhibit cardiac abnormalities that range from benign to more clinically significant. Cardiac abnormalities include incomplete right bundle branch block, left anterior hemiblock, shortened QT interval, ST segment elevation, and abnormal Q waves. However, reduction of the coronary vasodilative reserve, wall motion abnormalities, dilated cardiomyopathy, and heart failure have also been described (24).

Other forms of dystrophies may exhibit cardiac involvement less likely. For example, there is no clinically significant cardiac disease in FSH (25), other than sporadic cases of electrical and conduction problems, such as intraventricular conduction delay, supraventricular arrhythmias, ventricular tachycardia, atrioventricular block, long QT syndrome, and premature atrial contractions. Myocardial hypertrophy is much rarer and has been reported in one case. In Congenital Muscular Dystrophies, cardiac involvement is rare and has been only reported in the autosomal recessive FCMD with some reports of myocardial fibrosis and heart failure (26). In MD1, the main cause of morbidity and mortality is cardiac and respiratory involvement, and the heart and lungs can be affected even when muscular symptoms are mild. Cardiac involvement in MD1 almost always includes faulty impulse generation and propagation, such as atrioventricular block, prolonged QT, torsades de pointes, myocardial hypertrophy, trabeculation cardiomyopathy, and heart failure. There is also evidence that the coronary smooth muscles can be affected causing abnormal coronary flow reserve (27). In MD2, cardiac abnormalities include supraventricular tachycardia, ventricular ectopy, atrial fibrillation, heart failure, and myocardial infarction (27).

Finally, in patients with PROMM, cardiac involvement has been reported only in a few patients including sinus bradycardia, abnormal T waves, premature beats, right bundle branch block, and sustained monomorphic ventricular tachycardia (28).

#### Metabolic myopathies

There are at least 13 types of glycogenosis and the most clinically recognized ones include type 0 (Lewis' disease), type I (Von Gierke disease, the most common type, 90% of all glycogenosis), type II (Pompe's disease, acid maltase deficiency), type III (Cori's disease), type IV (Andersen's disease), type V (McArdle's disease), type VI (Hers' disease), type VII (Tarui's disease), type IX (phosphorylase kinase deficiency), and type XI (Fanconi-Bickel syndrome). Cardiac manifestations have only been described in glycogenosis type II, III, IV, VII, and IX. In type II ECG abnormalities, severe myocardial thickening and sudden death may occur. In type III, ECG abnormalities can be observed in up to 90% and myocardial hypertrophy occurs in up to 20% of patients. In type IV, myocardial hypertrophy, cardiomyopathy, and heart failure were reported (29). In type VII, chest pain characteristic of angina pectoris can present for years. Myocardial hypertrophy and abnormally speckled myocardium have also been reported (30).

Other forms of metabolic myopathies seem to exhibit cardiac involvement less frequently or in a more benign fashion (31). For example, in mitochondriopathies, cardiac involvement includes ECG abnormalities, myocardial thickening, dilatation, and heart failure (30). A dominant feature in patients with impaired respiratory chain enzymes seems to be left ventricular trabeculations.

The only but frequent cardiac involvement in very long-chain acetyl-CoA dehydrogenase deficiency seems to be myocardial thickening.

In patients with MADAD, myocardial thickening seems to be the exclusive cardiac manifestation in SCD, cardiac involvement comprises myocardial thickening exclusively.

Finally, in lysosomal storage diseases, myocardial thickening has been described. In females, myocardial thickening may be the only manifestation of the disorder. Severe cardiac muscle involvement may lead to early death already in infancy.

#### Disorders of the contractile proteins

Cardiac involvement here is rare. For example in Desmin myopathy atrial flutter, AV block and ventricular tachycardia can occur (32). Myocardial thickening, dilated myopathy, and systolic and restrictive diastolic dysfunction are also reported. In some cases, right ventricular dysfunction was the predominantly described cardiac abnormality. In CCD myocardial thickening, cardiac chamber dilatation, and heart failure, necessitating heart transplantation are reported in single cases (Tables 1, 2). As with other types of myopathies due to disorders of contractile proteins, cardiac involvement in nemaline myopathy (NM) is rare. However, all the above cardiac involvements are reported.

## Myopathies' effect on the lung function

Myopathies, in general, can affect the lung either primarily (intrinsic) or secondarily (extrinsic; Table 2, Figure 2) (33). Intrinsic effects can be direct lung parenchymal involvement which occurs independently of the type of myopathy causing decreased elastic lung properties and compliance (34). While poor lung maturity has been described in some of the myopathies, an insult to lung parenchyma can also develop by other mechanisms, such as chronic atelectasis due to hypoventilation and or chronic infections and aspiration pneumonia due to respiratory muscles and swallowing dysfunction that is associated with myopathy, which adds to the risk of developing respiratory failure. bronchiolitis, diffused alveolar hemorrhage (DAH), non-specific interstitial pneumonia (NSIP), usual interstitial pneumonia (UIP), or pulmonary fibrosis also occur in myopathic patients with similar mechanisms. The aforementioned parenchymal changes can lead to a decrease in total lung capacity, vital capacity, as well as expiratory reserve volume (33).

On the other hand, extrinsic effects are primarily due to weakness in primary respiratory muscles or associated thoracic cavity deformities The lung capacity decrease in this case independent of parenchymal changes when the involvement of the trunk muscles or chest wall deformities exist.

Other mechanisms that can cause respiratory involvement in patients with myopathy include excessive daytime sleepiness (EDS), which can occur in patients with myotonic dystrophy (DM1) in the absence of respiratory failure (35). The exact mechanism of EDS is not well understood; however, it is believed to be caused by central dysfunction of sleep regulation, and Modafinil, a central nervous system stimulant, has been used to manage it.

Bulbar weakness, which elevates the risk of developing aspiration pneumonia and obstructive sleep apnea, can also occur in patients with primary myopathies, such as in oculopharyngeal muscular dystrophy (36). Such mechanism is important to predict in patients who are specifically undergoing surgical procedures in whom intubations, tracheostomy, and prolonged intensive care may be required (37).

Another extra thoracic source of respiratory involvement can be observed in patients with lactic acidosis and stroke-like episodes, and myoclonic epilepsy (38), which can frequently complicate severe cases of metabolic myopathies. In that regard, respiratory involvement can be a result of secondary respiratory muscle weakness and central hypoventilation as a result of severe encephalopathy, or hyperventilation syndrome after lactic acidosis.

Overall, these patients have a reduced tidal volume and increased respiratory rate to try to compensate for their perception of dyspnea. As such, a final common outcome of all these mechanisms would be hypoventilation; however, such changes can lead to hypoxia, hypercapnia, or both, and an increased pulmonary artery resistance leading to inevitable pulmonary hypertension.

A clinically important aspect of this respiratory involvement of myopathies is that it is reportedly associated with unsuspected hypoxia that can be seen even in ambulatory patients. This hypoxia leads to hypoxic vasoconstriction, as a physiological response to avoid ventilation/perfusion mismatch. Chronic hypoxia affects the vascular tone and generates a certain degree of vascular remodeling and muscularization of the vascular media, leading to a subsequent increase in pulmonary vascular resistance. In conditions where the lung volumes are decreased such as in the myopathies with respiratory muscle involvement, there is a relative derecruitment of the vasculature that leads to a decrease in the vascular surface area aggravating the elevation in pulmonary vascular resistance. Chronic hypoxia also leads to secondary erythrocytosis that raises blood viscosity and thus further increases systemic and pulmonary vascular resistance.

Thoracic cavity deformities in addition to respiratory muscle weakness lead to extrathoracic lung restriction that can lead to changes in lung mechanics (39). There will be maximum inspiratory and expiratory pressures <30% of normal, vital capacity <55% of predicted, and total lung capacity <80% of predicted with a normal CO diffusing capacity. Poor lung mechanics can lead to hypercapnia and can also affect pulmonary function directly. Hypercapnia itself has less effect on pulmonary vascular tone compared to hypoxia. Vascular tone is mediated more due to the increase in hydrogen ion concentration and not due to the PaCO_2_ itself.

This sequala of the mechanisms explained above is pulmonary hypertension (40), leading to an increase in right ventricle wall stress and pressure overload that can lead to myocardial remodeling either in an adaptative way with minimal changes in the ventricle and arterial elastance producing a subtle clinical expression or a mal-adaptative remodeling in which dilation of the right ventricle and arrhythmias are more common with a more severe clinical presentation. It is pertinent to notice that in our case the patient had severely decreased static lung compliance pointing toward that the loss of pulmonary mechanics is not only related to extrapulmonary causes (muscle weakness) but possibly also related to intrapulmonary causes of poor lung distensibility. Myopathies can alter lung distensibility by unknown mechanisms, but some hypotheses have been described such as poor lung parenchyma maturation and chronic hypoventilation-induced–atelectasis or chronic micro-aspirations (41). In our patient, alveolar derecruitment and pulmonary edema are also intrapulmonary factors that alter intrapulmonary dispensability.

It is important to mention in this regard that NM is not classically associated with intrinsic effects and what is usually described as an extrinsic respiratory muscle involvement affecting both inspiration and expiration (42).

### Pulmonary manifestations of specific myopathies

Typical respiratory changes in myopathy can be found summarized in Table 1. The typical respiratory involvement in patients with DMD is a progressive increase in vital capacity as predicted until 10 years of age, after which it plateaus, then starts to fall thereafter with the development of respiratory muscle weakness and skeletal deformities (43). The rate at which vital capacity declines is around 8% per year (15). Serial measurement of vital capacity can monitor the progression of respiratory involvement and was shown to be a reliable predictor of mortality with a 5-year survival of 8% (16).

Respiratory impairment in myotonic dystrophy (DM1) manifests as a result of excessive daytime sleepiness (EDS), alveolar hypoventilation from a combination of respiratory muscle weakness, and dysfunction of the brain stem respiratory centers (44). Patients with DM1 are also at elevated risk of aspiration pneumonia, which has been shown in studies to be one of the most common causes of death in these patients.

Evaluation of respiratory status in patients with FSH is difficult as spirometry may be less accurate due to facial weakness causing poor mouth seals with mouthpieces. As such clinical assessment and arterial blood gas analysis are more helpful in these cases. A recent study suggested that about 1% of FSH patients required ventilatory support. And the most important predictors of significant respiratory involvement include disease severity, immobility, and deformities such as kyphoscoliosis (45).

In LGMD and EDMD, the development of scoliosis, contractures, and spinal rigidity can lead up to a restrictive pattern of respiratory impairment (46).

In metabolic myopathies, no reports of respiratory muscles being preferentially affected exist, although in mitochondrial encephalopathy, lactic acidosis, and stroke-like, and epileptic episodes can cause respiratory muscle weakness, sometimes with minimal limb involvement central hypoventilation or a hyperventilation syndrome after lactic acidosis (47, 48).

Finally, in myopathies due to disorders of the contractile proteins, respiratory involvement is inevitable in several types of the disease and can be early and fatal. In NM, however, the severity of respiratory involvement depends on disease onset and clinical severity. Marked respiratory involvement requiring early ventilation occurs in severe neonatal cases (29), while muscle weakness and respiratory involvement can be delayed to later in life in adult-onset NM, and can occur in ambulatory patients such as what was noted in the patient presented above (16).

## Myopathies' effect on the cardiopulmonary interaction

Myopathies can disrupt cardiopulmonary coupling by affecting interactions between cardiac diastolic and systolic, and respiratory functions, as well as disruption of ventilation–perfusion relationships (Table 2, Figure 2).

When lung mechanics are affected as in the case of our patient, an extra-thoracic restriction develops that subsequently impairs the cardiac function. Patients with either intra or extrathoracic lung restriction have to generate a more negative intrapleural pressure to create a close to normal tidal volume due to its relatively stiff chest wall. As in patients with obstructive sleep apnea and COPD, the repetitive periods of airway obstruction, there is increased respiratory effort in a trial to increase respiratory drive and correct minute ventilation. This increased respiratory effort will result in marked changes in intrathoracic pressure during inspiration and expiration. The exaggerated decreased intrathoracic pressure during inspiration and the subsequent increased intrathoracic pressure during expiration reduces venous return, and cardiac transmural filling pressure, and thus reduces right ventricular output (49).

Moreover, marked changes in intrathoracic pressure compress the heart within the thoracic cavity, which is exaggerated if the lungs are hyperinflated, such as in obstructive airway diseases, and such elevated pressures get transmitted to the pulmonary vasculature and ventricular walls increasing the tension, causing diastolic dysfunction and activating the sympathetic system causing more salt and water retention leading to hypertension and left ventricular hypertrophy that can later progress into both diastolic and systolic heart failure (50).

Finally, this cardiopulmonary disruption leads to myopathy-related V/Q mismatch causing hypoventilation, hypercapnia, and hypoxemia. It is to be noted that respiratory muscle weakness alone is sufficient to account for hypercapnia when respiratory muscle strength is <30% of normal. However, the addition of cardiac and pulmonary elements observed in myopathic patients as described above can aggravate hypercapnia and hypoxia beyond expected levels.

### Management of myopathy

There are few specific therapies to correct the cardio-pulmonary effects of myopathies. Treatments always follow guidelines for general involvement in the absence of myopathy. Early detection and management are important for favorable outcomes. Along the same lines, the management of cardio-pulmonary involvement of Nemaline myopathy is also mostly general management with few options that can be considered myopathy-specific management (51).

### Management of cardiac involvement

Early detection and management of cardiac involvement are important for favorable outcomes. Most of the therapies follow the same guidelines directed to management for general cardiac involvement. Despite so, and because of the confusion that always accompanies the options for the management of myopathic patients, the American heart association has recently developed a road map for management of patients with cardiac involvement in myopathy (31). As such, all myopathic patients, regardless of genotype, should be referred for cardiology assessment at the time whether they are symptomatic or not and at least an annual evaluation with an echocardiogram, ECG, and ambulatory ECG is reasonable.

Regardless of the type or clinical status, poor cardiorespiratory endurance is common in myopathic patients, and despite so, the role of physical training and exercise in patients with myopathies is unknown and at best only associated with modest clinical improvement (31).

Some preclinical options of treatment exist under development and are still in clinical trials. These therapeutic options, however, superficially target the correction of the skeletal muscle compromise. Examples include utrophin up-regulation, stop codon read-through therapy, viral gene therapy, cell-based therapy, or exon skipping (52). Therapies focused on the myocardium, however, do not exist.

Some myopathy-specific medications may have beneficial effects on cardiac involvement. For example, steroids can be used in DMD patients and may be effective in delaying the onset of cardiomyopathy (6, 53, 54). Enzyme replacement therapy (ERT) can be beneficial in Pompe's disease complicated with hypertrophic cardiomyopathy (55). ERT can be also beneficial in neonates and pediatric cases with cardiac involvement (56). Administration of L-carnitine in carnitine deficiency may have a stabilizing effect on cardiomyopathy as well (57).

General specific measures include avoidance of cardiotoxic drugs, conservative guidelines directed medical therapies for heart failure therapy, including angiotensin-converting enzyme inhibitors, angiotensin receptor blockers, beta-blockers, diuretics, aldosterone-antagonists, Niprylisin inhibitors, and sodium–glucose transport 2 (SGLT2) inhibitors, as well as ICDs and cardiac resynchronization therapy (58).

In cases of atrial fibrillation with or without systolic dysfunction, oral anticoagulation should be started, and cardioversion and ablation options can be considered. ICDs should be used in patients with non-sustained ventricular tachycardia who may benefit from beta-blockers (58).

Pacemakers can be also lifesaving in patients with bradycardia and conduction blocks. In patients with myocardial ischemia with suspected coronary disease, coronary angiography, and stenting if applicable followed by antiplatelets and antithrombotic treatment is indicated.

### Management of respiratory involvement in myopathy

Early detection is key for the effective management of respiratory problems with myopathy. Asymptomatic patients should have respiratory muscles assessed at least once using vital capacity and spirometry pressures to establish subclinical disease and baseline numbers in non-affected patients. If respiratory muscle involvement is well established, annual spirometry should be performed to assess for progression (59).

In symptomatic patients, treatment can start with respiratory muscle training. Few clinical trials concerned with respiratory muscle training reported that strength or endurance exercises show some benefit in these patients; however, long-term clinical benefit is uncertain (60, 61).

Other techniques that can be used in symptomatic patients to improve cough and prevent aspiration have been described. Such maneuvers include manual compression, glossopharyngeal (frog) breathing, and insufflation using a self-inflating resuscitation bag, as well as mechanical cough-assist devices (62).

Noninvasive ventilation (NIV) *via* a mask can be started intermittently in these patients at an earlier stage. The appropriate time to start NIV in myopathic patients, however, remains controversial as prophylactic use had been shown to be linked to increased mortality in some patients with specific myopathies, such as DMD (63). As such, NIV is only recommended if impending respiratory failure is expected, such as in patients with daytime hypercapnia (64), while in patients with severe respiratory failure, it is safer to intubate them than to use NIV, followed by a trial of extubating toward NIV, that is preferable over tracheostomy, which is only reserved classically to patients who become NIV or invasive ventilation dependent.

Needless to say, all patients with myopathy should have primary prevention for common infections, such as pneumococcal, influenza, and COVID-19 infections. Antibiotics to treat respiratory infections should also be handy. In patients with bulbar symptoms, speech and language therapy is also advised to reduce the risk of aspiration.

## Nemaline myopathy: From clinical suspicion to cardiopulmonary management

NM, the disease in the illustrative case, is a rare type of congenital myopathy causing disorders of the contractile proteins that can be either autosomal dominant or recessive with an incidence of 1 in 50,000. NM often exhibits hypomyotonia and muscular weakness. It is characterized by the presence of rod-like structures (positive red-stained with Gomori trichrome method) in the skeletal muscle fibers or the presence of Nemaline bodies in electron microscopy.

A large variety of gene mutations has been described in NM as mentioned earlier (5). The most recognized are mutations in the slow α-Tropomyosin Gene (TPM3) that causes muscle weakness predominantly in the lower limbs and affects mostly type 1 fiber. Mutations in the Nebulin Gene (NEB) cause more axial muscle weakness than upstream limb weakness. Most of these patients are unable to lift their heads and have nasal voices and dysarthria. Mutations in the skeletal muscle α-Actin Gene (ACTA1), gives usually a severe manifestation of the disease with the neonatal presentation of a floppy infant with failure to breathe. There are many other mutations associated with NM, which give specific pictures and characteristic symptoms.

Patients with the congenital typical form of NM, initially present with upstream, and later downstream progressive muscle weakness (4). NM is clinically heterogeneous, and at least 6 types of clinical manifestations have been described (5). NM types were classified according to the form of NM, ambulation, the requirement for home mechanical ventilation (HMV), and spinal deformity into: (1) severe NM; which is characterized by contractures, fractures, lack of respiratory effort, or severe hypotonia with lack of movements at birth; (2) typical NM: which is characterized by having a perinatal onset and delayed motor milestones delayed; (3) mild NM: which is characterized by being childhood or juvenile onset; (4) distal (downstream) NM: a form of NM that is mainly characterized by distal (downstream) muscle weakness; (5) childhood-onset NM with slowness: characterized by sluggish movements and core-rod histology (*KBTBD13* mutation); and (6) recessive *TNNT1* (former Amish) NM. It is clear that in the case of our patient he had the typical congenital form with a progressive slow course.

Importantly, a combination of acute or chronic heart failure associated with acute or chronic respiratory failure can occur in any form of NM that survive beyond childhood, despite being rare, and could be attributed to either cardiac or respiratory affections. A mixed cardiopulmonary involvement is rare in NM, especially when other etiologies for respiratory and heart failure had been ruled out as illustrated in our case.

In a recent review of literature for causes of sudden cardiac death in Nemaline myopathy (53), types and frequencies of cardiac involvement were identified in 35 patients. Of these patients, 22 were male and 8 were female. The disease was neonatal onset in 16, infantile-onset in 5, and adult-onset in 4. Nine of these patients presented with dilated cardiomyopathy, six with hypertrophic cardiomyopathy and one with nonspecific cardiomyopathy. Moreover, the ventricular septal defect was present in two patients. Four of these patients developed heart failure, and 1 had sudden cardiac death. Long QT syndrome has also been reported in patients with NM, and conduction disturbances are also common.

The mechanisms of cardiac involvement in patients with NM, as with all other forms of myopathies, is heterogenous, and include cardiac myocyte dysfunction, infiltrative process, coronary endothelial dysfunction, and myocardial fibrosis. As such, left and right ventricular failure myocardial ischemia and infarction, as well as arrhythmias. The RV failure as well as lactic acidosis developing due to cardiogenic shock and low cardiac output states may affect respiratory functions and pulmonary vascular tone and pressure.

Importantly, that the patient presented above had ECG changes and echocardiographic evidence that may suggest myocardial ischemia. While the patient refused to peruse further evaluation for coronary disease, other mechanisms such as coronary endothelial dysfunction and stress-induced cardiomyopathy may be possible explanations.

**Figure 3:**
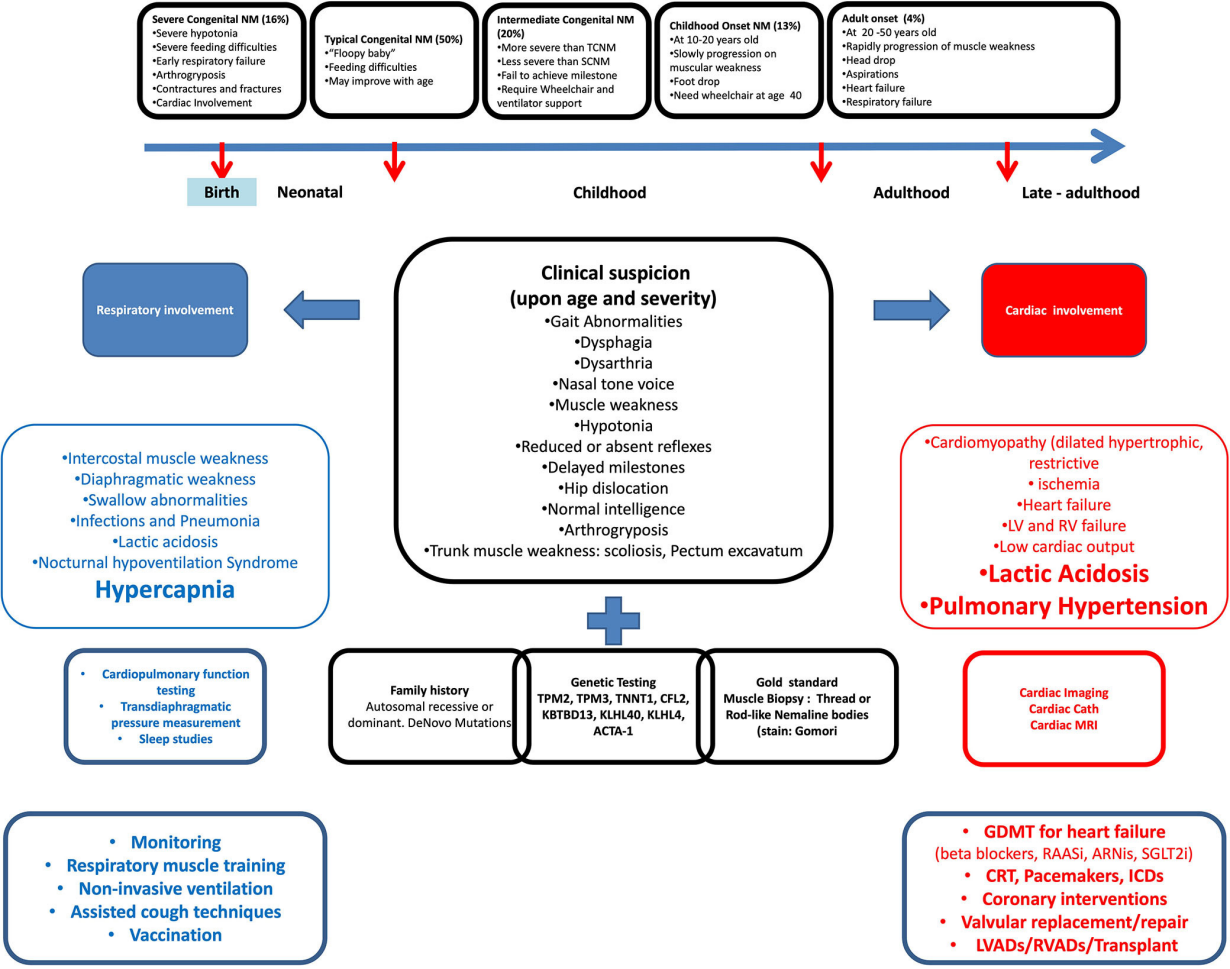
Cardiopulmonary involvement in nemaline myopathy from clinical presentation to management.

As with all other myopathies, all aforementioned conventional cardiac workups and therapies are indicated in NM patients (Figure 3).

On the other side, respiratory involvement in NM patients is common. The main mechanism seems to be mainly related to intercostal and diaphragmatic muscle weakness. However, swallow abnormalities associated with lung infections, nocturnal hypoventilation syndrome, and decreased lung mechanics due to arthrogryposis and trunk muscle weakness are also reported.

In one study, all NM patients reported respiratory symptoms regardless of the degree of respiratory muscle weakness.

Reportedly, there is a low correlation between motor function and spirometry values in patients with NM, which confirms an observation made in other myopathic patients that the degree of motor function impairment is not representative of respiratory muscle function and may be related to the heterogeneity in the genotypes. Importantly, in patients with NM, if the respiratory muscles are involved, hypoventilation and pre-capillary pulmonary hypertension may occur because of hypoxia or primary airway involvement that can secondarily cause strain on the right side of the heart as seen in our patient.

As such, early identification of respiratory muscle weakness is desirable, given the availability of therapies that have been shown to improve survival and quality of life (65). As such screening as well as follow-up cardiopulmonary testing with spirometry, sleep studies for assessment of nocturnal hypoventilation, as well as pulmonary imaging may be needed for initial assessment as well as for monitoring of progression.

Once a diagnosis of respiratory muscle weakness has been made, supportive respiratory therapy should be initiated. The approach to management is similar to respiratory management in other conditions and other forms of myopathy (66) including NIV, cough assist, lung-volume recruitment, and respiratory muscle training. Follow-up of NM patients that have early respiratory muscle weakness who do not require therapy should be decided based on the clinical suspicion of progression and that in patients requiring therapy should be done at least every 3 months or more frequently if new therapies are being started.

As such, NM harbors several mechanisms that are to affect these cardiopulmonary coupling greatly, as can be seen in our patient who had multiple mechanisms of cardiac impairment as evident by cardiomyopathy and myocardial strain, and respiratory impairment characterized by respiratory muscle weakness and disruption of neuronal mechanisms.

## General phenotypic description

Heart-lung disease (HLD) is a heterogeneous condition resulting from loss of CPC with any or combinations of its various mechanisms. As such HLD can manifest as either a pulmonary or a cardiac condition. Pulmonary HLD (type I cardiopulmonary disease) is a condition where hypoxia, hypercapnia, respiratory failure, and dyspnea will predominate (67). In this type, the respiratory function seems to be impaired and ECG may not show RV strain or right axis deviation and echocardiogram shows preserved heart function with preserved TAPSE and Right ventricle function. Cardiac HLD (type 2 cardiopulmonary disease) occurs when cardiac impartment is more prominent and symptoms and signs of heart failure, as well as ischemia, are notorious with ECG suggestive of LVH and right ventricle dysfunction however the respiratory reserve is usually mildly impaired.

**Figure 4:**
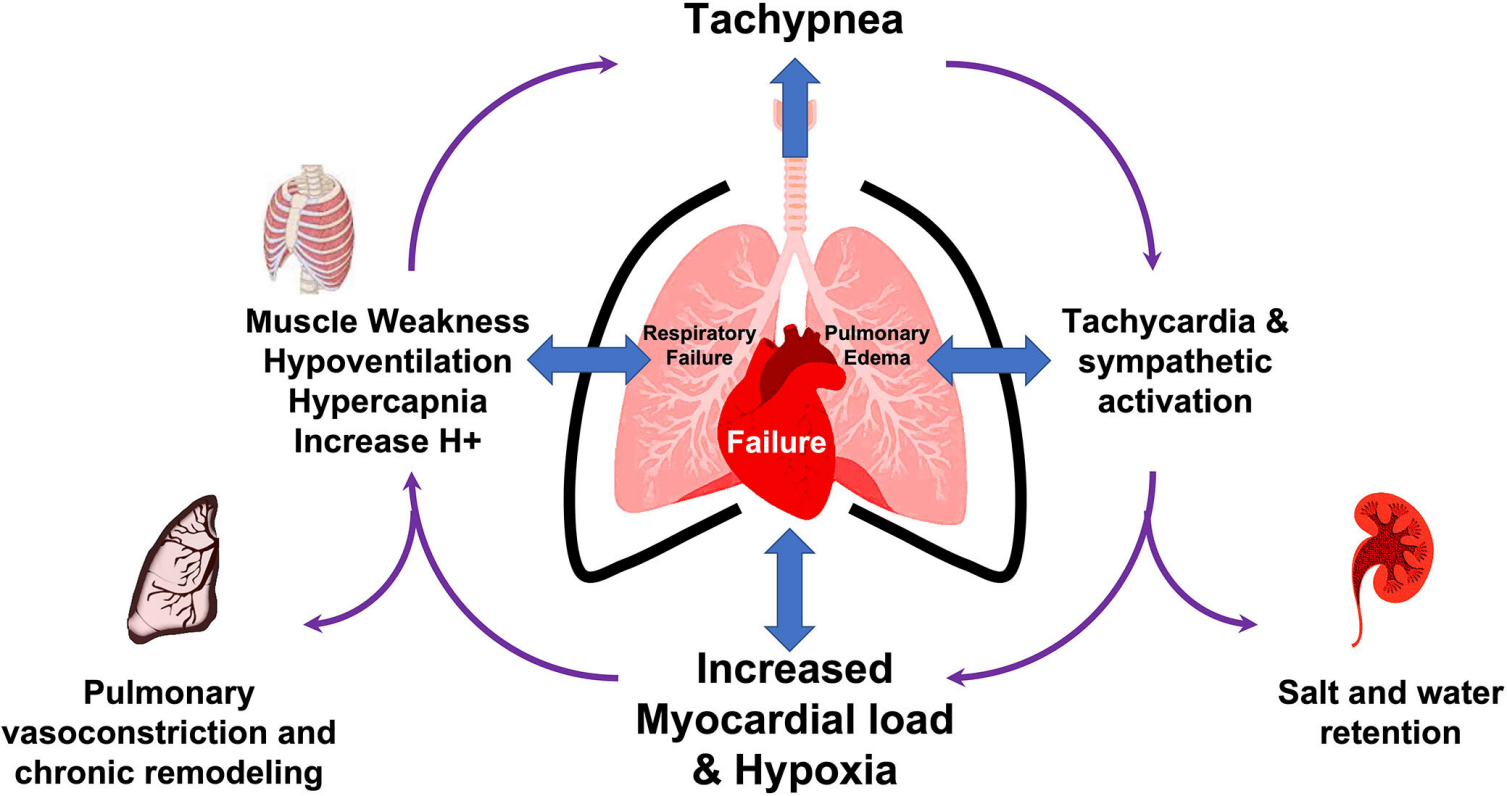
Summary of mechanisms of disruption of cardiopulmonary coupling in the illustrative case.

In our patient, there are multiple mechanisms by which CPC was altered (Figure 4). First, the patient had decreased ejection fraction together with ST changes that suggest myocardial ischemia, inflammation, or at least a level of myocardial strain that can be exerted through elevated pressures, all of which can happen as a result of the myopathic nature of his congenital NM. As such, a cardiopulmonary mechanical coupling initiated from the left side of the heart with resultant backward effect and pulmonary congestion can be noted. Moreover, the inherent nature of NM leads to an expected respiratory muscle weakness leading to hypoventilation which seems to add further pathological layer initiated from the pulmonary side and aggravate the cardiopulmonary uncoupling. To some extent, this may have also led to disruption of the neuronal control of both respiration and heart rate despite near normal blood pressure and leading to a triple effect of hypoxia/hypercapnia/tachycardia. As such, clinical improvement was not possible with mechanical ventilation alone and the management required diuresis for volume management as well as mechanical ventilation for support of respiratory muscle and gas exchange. With these interventions at multiple levels of cardiopulmonary interaction, the clinical recovery was rapid such that in 24 h the patient was able to be discharged from the ICU.

As such, this patient does not fit into a primary cardiac or a primary pulmonary type of HLD and a third phenotype seems to be necessary for an appropriate description of the pathophysiological consequences. An appropriate nomenclature for this third phenotype can be described as a mixed cardiopulmonary disease in which neither the pathological process nor the management plan should focus on either side of CPC rather efforts should be paid to diagnosis and management at the lung and the heart simultaneously.

## Conclusions

The heart and the lung are two organs that function in a physiologically coupled fashion at multiple levels in what is known as cardiopulmonary coupling (CPC). Heart–lung disease is a manifestation of the loss of the CPC with any mechanism in isolation or combination resulting in a cascade of neuronal, mechanical, and pathological events that lead to cardiopulmonary failure. HLD can be initiated with pulmonary pathology (type 1 cardiopulmonary disease) or with cardiac pathology (type 2 cardiopulmonary disease). Primary skeletal muscle myopathy is a group of conditions that can disrupt CPC at both levels. Nemaline myopathy specifically is a rare condition that has pathological effects that can be linked to respiratory as well as cardiac pathology that can progress with age to levels beyond previously identified. The recognition of a type of cardiopulmonary disease that can potentially simultaneously affect the heart and the lung calls for a framework in which the two previously described phenotypes (type 1 HLD and type 2 HLD) are combined with the specific third phenotype of (type 3 HLD and mixed cardiopulmonary disease) explained in this article, the recognition of which can aid the early diagnosis for this poorly characterized HLD in the mixed bag of diseases primary myopathy is.

## Author contributions

DR-B: case study material preparation, literature review, and manuscript write up submission. RS: review of literature manuscript preparation and supervision. AD and LY: manuscript preparation and submission. All authors contributed to the article and approved the submitted version.

## Conflict of interest

The authors declare that the research was conducted in the absence of any commercial or financial relationships that could be construed as a potential conflict of interest.

## Publisher's note

All claims expressed in this article are solely those of the authors and do not necessarily represent those of their affiliated organizations, or those of the publisher, the editors and the reviewers. Any product that may be evaluated in this article, or claim that may be made by its manufacturer, is not guaranteed or endorsed by the publisher.
