# Supplementary material for: Disruption of cardio-pulmonary coupling in myopathies: Pathophysiological and mechanistic characterization with special emphasis on nemaline myopathy
Source: Front Cardiovasc Med. 2022 Nov 7;9:996567. doi: 10.3389/fcvm.2022.996567 (PMC9676365; doi:10.3389/fcvm.2022.996567)
Supplement: Supplementary file 1 [file Table_1.DOCX]

**Disruption of Cardio-Pulmonary Coupling in Myopathies: Pathophysiological and Mechanistic Characterization with Special Emphasis on Nemaline Myopathy**

Diana Maria Ronderos Botero MD, Arundhati Dileep MD, Laura Yapor MD,

Ravish Singhal MD

**Author affiliation**

Division of Pulmonary and Critical Care Medicine, Department of Medicine, BronxCare Health System, Bronx, USA

**Author of correspondence:**

Diana Maria Ronderos Botero

Division of Pulmonary and Critical Care, Departent of Medicine,

1650 Grand Concourse BronxCare Hospital Center, Bronx, NY, USA

Phone number: +1(347)342-2581

Email: [dronderos@bronxcare.org](mailto:dronderos@bronxcare.org)

**Supplementary material**

**Cardiorespiratory coupling (CRC)**

The reciprocal nature of the co-expression of arterial pulse and respiratory rhythms indicates, at least, a middle ground of coupled control[1]. Cardiorespiratory coupling (CRC) result from shared complementary functions affecting heart rate (HR), blood pressure (BP), and ventilation rhythms[2, 3]. These factors can be categorized into neuronal factors, reflex factors, central factors, and chemoreceptors[4].

Neural factors affecting the CRC are directly coupled to autonomic regulation. The neural activity increases sympathetic discharge during inspiration and vagal discharge during expiration leading to an increase in heart rate during inspiration and a decrease during expiration or what is known as respiratory sinus arrhythmia. However due to the rapid deactivation of acetylcholine in parasympathetic fibers and the significantly slower deactivation of norepinephrine in the sympathetic fibers, respiratory sinus arrhythmia is almost exclusively a vagal response.

Reflex factors affecting the CRC are also tightly related to autonomic regulation through stretch receptors, and baroreceptors (supplement figure 1A)[5]. Stretch receptors in the lungs are stimulated during inspiration leading to a reflex increase in heart rate through vagal reflex mechanisms[6]. The other type of stretch receptors affecting this process exist in the right atrium. Inspiration decreases intrathoracic pressure thereby increasing venous return to the right side of the heart leading to a consequent stretch of the right atrial stretch receptors causing an increase in heart rate in what is known as the Bainbridge reflex[7]. After the time delay required for the increased venous return to reach the left side of the heart, left ventricular output increases and raises arterial blood pressure in what is known as Traube–Hering waves[8]. This rise in blood pressure in turn reduces the heart rate through the baroreceptor reflex[9].

Central factors responsible for respiratory sinus arrhythmia reside in the medulla through the cardiac autonomic centers (supplement table 1, supplement figure 1) and their interaction with the respiratory center. These are seen in animal models independent of the lungs itself as it was found that in heart-lung bypass preparations that when the chest was opened and the lungs were collapsed while venous return was diverted to a pump-oxygenator at a constant BP, rhythmic movement of the rib cage attests to the activity of the medullary respiratory centers and were accompanied by rhythmic changes in HR at the respiratory frequency[10, 11]. This highlights a direct interaction between respiratory and cardiac centers in the medulla that are independent of reflex or neuronal activities.

Finally, the cardiovascular system responds to [peripheral chemoreceptor](https://www.sciencedirect.com/topics/medicine-and-dentistry/peripheral-chemoreceptors) stimulation illustrating another level of complexity[12]. Carotid sinus [chemoreceptors](https://www.sciencedirect.com/topics/medicine-and-dentistry/chemoreceptor), for example, increases ventilatory rate and depth as well as changes the heart rate. It is the resultant ventilatory changes magnitude that determines changes in heart rate as a result of carotid [chemoreceptor](https://www.sciencedirect.com/topics/medicine-and-dentistry/chemoreceptor) stimulation such that mild chemoreceptor stimulation of respiration decreases the heart rate moderately and more pronounced stimulation increases the heart rate only slightly. At the extreme end, if the chemoreceptor-ventilation stimulation is blocked, the heart rate response becomes maximal (supplement table 1, supplement figure 1).

One can conclude that CPC is a physiological process that functions as a result of a shared neuronal triggered responses initiated at the respiratory levels (direct autonomic effects), or reflex delayed response (baroreceptors, carotid sinus sensory activity, and chemoreceptors), reflex responses (respiratory and atrial stretch receptors), and central responses (medullary cardiac-respiratory center interaction). However, most of these factors are debated to have only a weak role in resting state. In fact, the relative effects of each of these mechanisms on the overall cardiopulmonary function is still a matter of debate. For example, studies have shown little or no effect of the CPC in patients with cardiac pacemakers with stable fixed HR. However, other recent studies suggest that the delayed response has a bigger role in this relationship rather than an interaction between brainstem neural networks

**Cardiopulmonary mechanical coupling (CPMC)**

As with neuronal mechanisms, mechanical coupling as well can be appreciated in both heart and lungs. From the cardiac standpoint, cardiac and pulmonary interactions occur at both the left side and the right side. The left heart is related distally to the systemic circulation, and proximally to the right side of the heart through the pulmonary veins as such left sided pressures reflect to the pulmonary circulation and any pathological increase in these pressures from the ventriculo-aortic level to valvular heart disease and cardiomyopathies will result in a backward increase in pulmonary pressures leading to pulmonary edema and right sided heart failure[13, 14]. On the other hand, the right heart is related distally to the pulmonary circulation and proximally to venous return as such pathological alterations in the lung parenchyma or vasculature can affect the right side leading to elevated pressures and venous hypertension and edema (supplement table 1, supplement figure 2)[14].

From the pulmonary perspective, changes in respiratory mechanics significantly impact pulmonary hemodynamics and ventricular filling and ejection. Such mechanical coupling is a less recognized factor that can affect HR and BP in addition to neuronal mechanisms. The anatomical location of the lungs and heart in the tightly sealed and isolated thoracic cavity results in free transmission of intrathoracic pressures towards the heart and the lungs affecting ventilation, perfusion, venous return, and cardiac pumping.

Inspiration decreases thoracic pleural pressure which is transmitted to all cardiac chambers resulting in increase in venous return, increasing HR and right sided cardiac output to the expanded elastic lung that during inspiration accommodates more blood volume and as such decreases venous return to the left side of the heart leading to a slight drop of left sided cardiac output. The reverse occurs during expiration[15]. This phenomenon when noticed at the ventricular level leads to slight expansion of the right ventricle on the expense of the left ventricle during inspiration and its reverse during expiration, or the so called “ventricular interdependence” the exaggeration of which occurs in some pathologies such as constrictive pericarditis and cardiac tamponade[16]. Such phenomenon also leads to slight variation in systolic blood pressure (<10 mmHg drop during inspiration), the exaggeration of which in pericardial diseases is clinically known as pulsus paradoxus[17].

***Summary:*** CPC is governed by a) respiratory and hemodynamic neuronal, reflex, central and chemical mechanisms that control rhythm and rate of respiration as well as HR and BP, b) mechanical coupling at the level of ventricular interdependence that is affected by changes in the thoracic pressure during respiration, and c) connections of the right heart to the precapillary component of the pulmonary circulation and the left heart to the post capillary component of the pulmonary circulation. These factors add complex mechanisms by which CPC can mediate ventilation, perfusion, cardiac diastolic and systolic performance.

It’s to be noted that changes in intrathoracic pressures are tightly related to healthy respiratory muscular contractions. As such, myopathy is expected to affect these mechanisms greatly, as can be seen in our patient with Nemaline myopathy who had multiple mechanisms affecting CPC; he had cardiac impairment as evident by cardiomyopathy and myocardial strain, and respiratory impairment characterized by respiratory muscle weakness and disruption of neuronal mechanisms.

**References:**

1 Koeppen BM, Stanton BA. Regulation of the Heart and Vasculatur *Berne and Levy Physiology, Seventh Edition* 2018:386-409.

2 Bernardi L, Rossi M, Soffiantino F, et al. Cross correlation of heart rate and respiration versus deep breathing. Assessment of new test of cardiac autonomic function in diabetes. *Diabetes* 1989;**38**:589-96.

3 Grossman P, van Beek J, Wientjes C. A comparison of three quantification methods for estimation of respiratory sinus arrhythmia. *Psychophysiology* 1990;**27**:702-14.

4 Downey JA, Myers SJ, Gonzalez EG, et al. Chapter 7 - Cardiopulmonary Physiology. *The Physiological Basis of Rehabilitation Medicine (ed 2)* 1995:127-47.

5 Llewellyn-Smith IJ, Verberne AJM. Central Regulation of Autonomic Functions. *Oxford University PressNew York* 2011.

6 Bianchi AL, Denavit-Saubie M, Champagnat J. Central control of breathing in mammals: neuronal circuitry, membrane properties, and neurotransmitters. *Physiol Rev* 1995;**75**:1-45.

7 Hakumaki MO. Seventy years of the Bainbridge reflex. *Acta Physiol Scand* 1987;**130**:177-85.

8 Barnett WH, Latash EM, Capps RA, et al. Traube-Hering waves are formed by interaction of respiratory sinus arrhythmia and pulse pressure modulation in healthy men. *J Appl Physiol (1985)* 2020;**129**:1193-202.

9 Timmers HJ, Wieling W, Karemaker JM, et al. Cardiovascular responses to stress after carotid baroreceptor denervation in humans. *Ann N Y Acad Sci* 2004;**1018**:515-9.

10 Guyton AC. Determination of cardiac output by equating venous return curves with cardiac response curves. *Physiol Rev* 1955;**35**:123-9.

11 Levy MN. The cardiac and vascular factors that determine systemic blood flow. *Circ Res* 1979;**44**:739-47.

12 Dempsey JA, Smith CA. Update on Chemoreception: Influence on Cardiorespiratory Regulation and Pathophysiology. *Clin Chest Med* 2019;**40**:269-83.

13 Janicki JS, Weber KT, Likoff MJ, et al. The pressure-flow response of the pulmonary circulation in patients with heart failure and pulmonary vascular disease. *Circulation* 1985;**72**:1270-8.

14 Maron BA, Kovacs G, Vaidya A, et al. Cardiopulmonary Hemodynamics in Pulmonary Hypertension and Heart Failure: JACC Review Topic of the Week. *J Am Coll Cardiol* 2020;**76**:2671-81.

15 Wise RA. Effect of alterations of pleural pressure on cardiac output. *South Med J* 1985;**78**:423-8.

16 Naeije R, Badagliacca R. The overloaded right heart and ventricular interdependence. *Cardiovasc Res* 2017;**113**:1474-85.

17 Sarkar M, Bhardwaj R, Madabhavi I, et al. Pulsus paradoxus. *Clin Respir J* 2018;**12**:2321-31.

**Figure legends:**

**Supplementary figure 1:** Cardiopulmonary neuro coupling mechanisms. A, neural regulation for production of respiratory sinus arrhythmia (RSA). RSA is regulated by neuronal effects, central interaction between the respiratory and cardiac centers in the medulla, as well as by reflexes due to stretch receptors in the lungs and the right atrium (the Bainbridge reflex), as well as from baroreceptors in the carotid sinuses and aortic arch. B, Cardiopulmonary effects of chemoreceptors. Activation of peripheral chemoreceptors stimulates the medullary cardiac inhibitory center leading to decrease in heart rate. Peripheral chemoreceptor stimulation, on the other hand, activates medullary respiratory center causing hypocapnia and lung inflation and stretch, both of which secondarily inhibit the medullary vagal center and indirectly attenuate the primary reflex effect of peripheral chemoreceptor on heart rate.

**Supplementary figure 2:** Cardiopulmonary mechanical coupling

**Supplementary figure 3:** Ventilation perfusion relationships and causes of dead space and shunting

**Supplement table 1: Cardiopulmonary interactions: types, definitions, mechanisms, and effects**

| Mechanism | Definition | Mechanisms | Effects |
| --- | --- | --- | --- |
| Cardiorespiratory coupling (CRC) | shared complementary functions between the heart and the lung affecting heart rate, blood pressure, and ventilation rhythms initiated at the respiratory side | Neuronal | Changes in HR and BP secondary to changes in respiration |
|  |  | Reflex | HR: increase heart rate during inspiration by stretch receptors in the lung and in the right atrium (bainbridge reflex)  BP: BP increase during inspiration (Traube–Hering waves). |
|  |  | Central | Interactions between respiratory and cardiac centers in the medulla independent of the lungs |
|  |  | Chemoreceptors | Carotid sinus [chemoreceptors](https://www.sciencedirect.com/topics/medicine-and-dentistry/chemoreceptor) stimulation increases ventilatory rate and depth |
| Cardiopulmonary mechanical coupling (CPMC) | Cardiopulmonary | Precapillary pulmonary vasculature | Right ventricular failure, Elevated pulmonary pressures and venous hypertension and edema in patients with primary right sided diseases or pulmonary bronchial or parenchymal diseases |
|  |  | Post capillary pulmonary circulation | backward increase in pulmonary pressures all the way to pulmonary edema and right sided heart failure that occur due to left heart disease |
|  | Pulmonary-Cardiac | ventricular interdependence | slight expansion of the right ventricle on the expense of the left ventricle during inspiration and its reverse during expiration |
|  |  | pulsus paradoxus | variation in systolic blood pressure during inspiration |
