# Supplementary figures and images for: Disruption of cardio-pulmonary coupling in myopathies: Pathophysiological and mechanistic characterization with special emphasis on nemaline myopathy
Source: Front Cardiovasc Med. 2022 Nov 7;9:996567. doi: 10.3389/fcvm.2022.996567 (PMC9676365; doi:10.3389/fcvm.2022.996567)

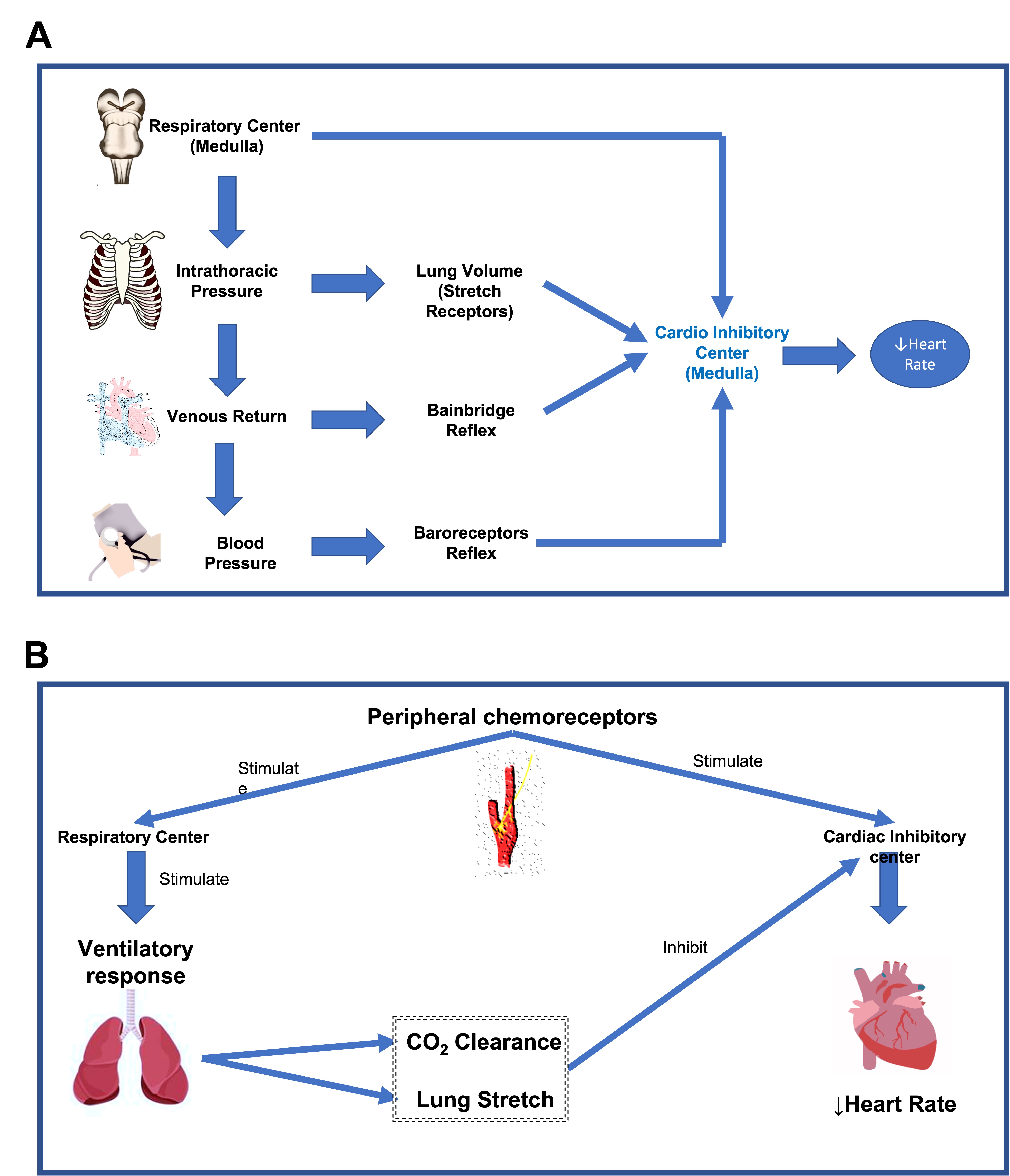

Supplement: Supplementary file 2 [file Image_1.JPEG]

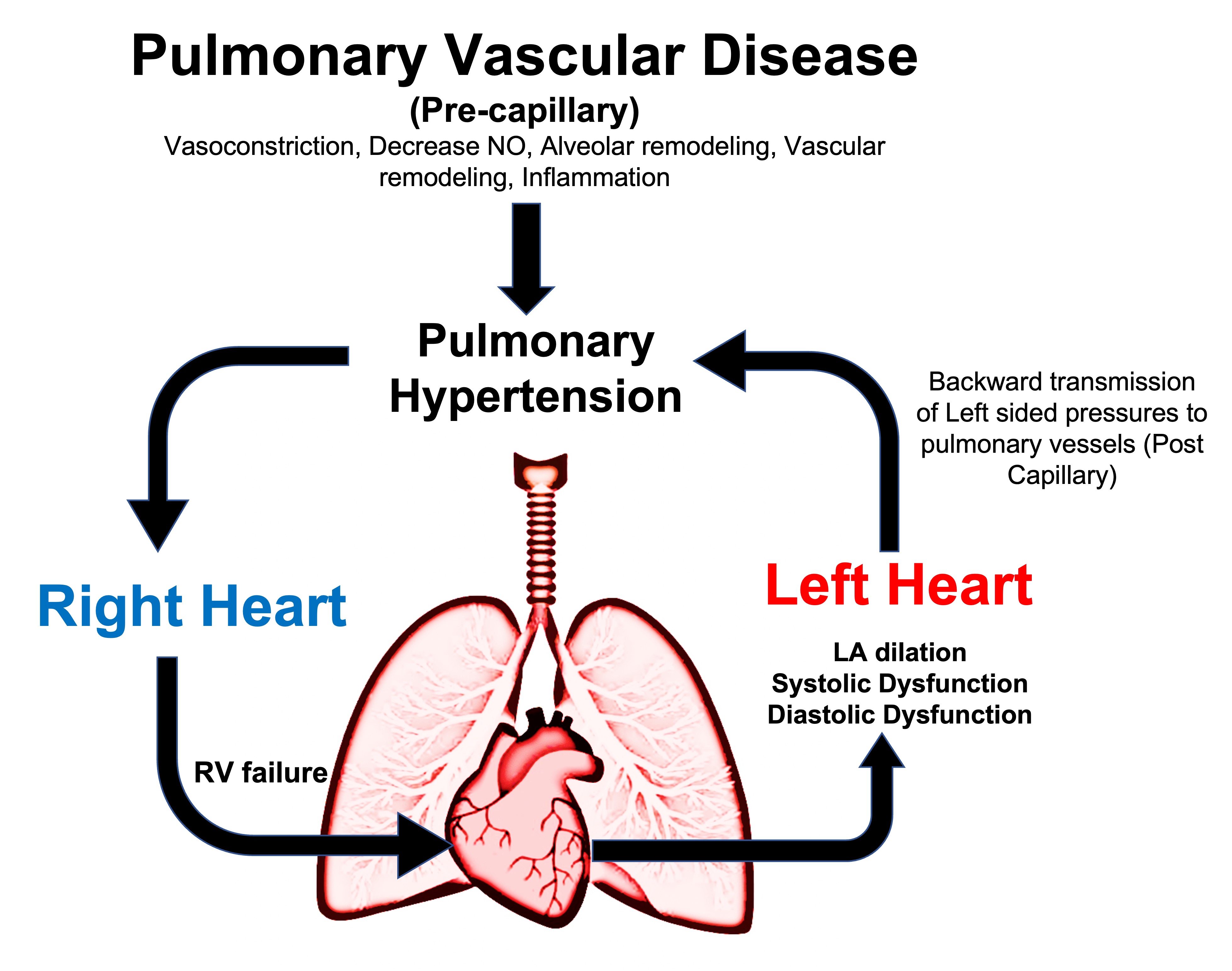

Supplement: Supplementary file 3 [file Image_2.JPEG]

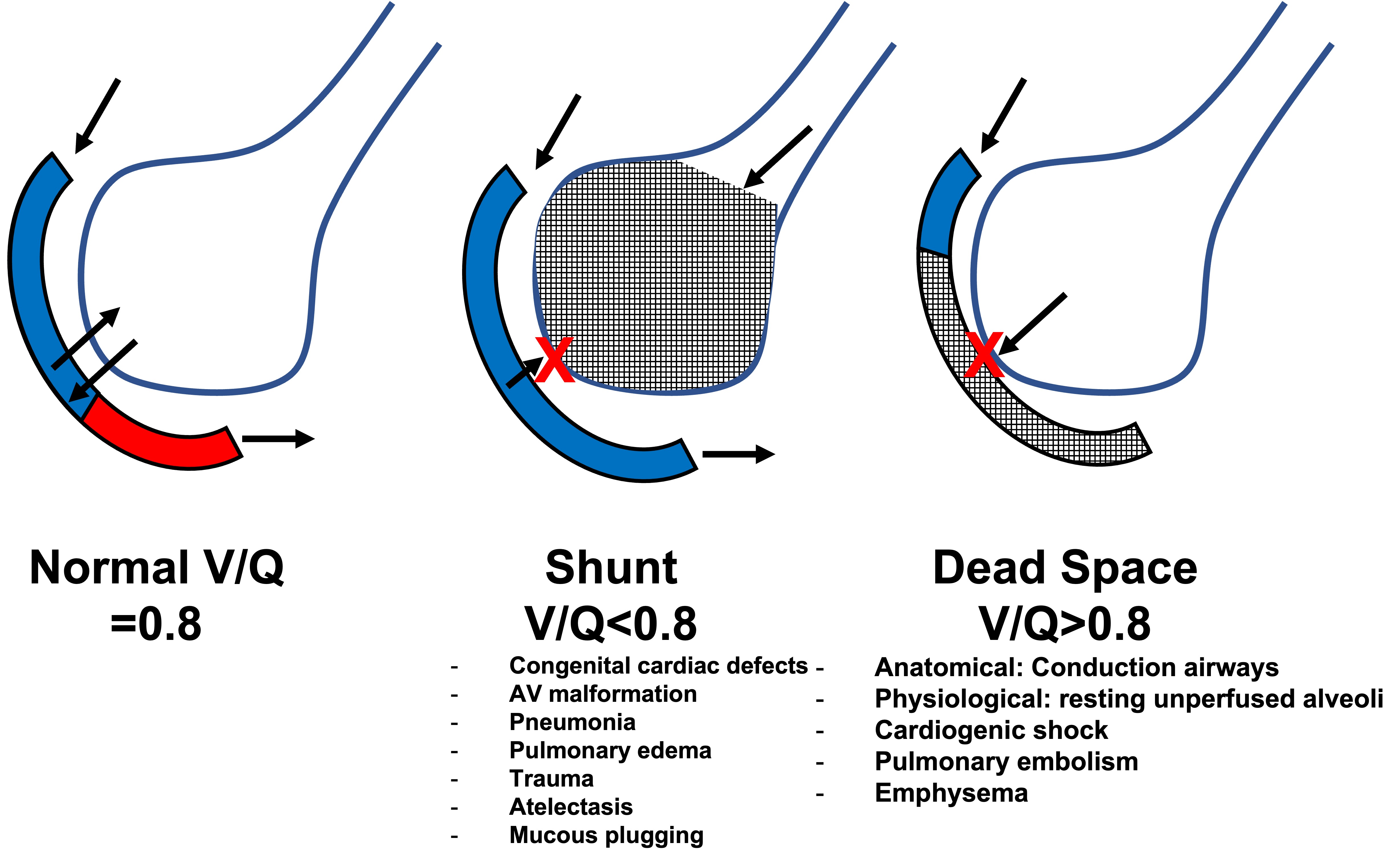

Supplement: Supplementary file 4 [file Image_3.JPEG]
